# Metabolism of HSAN1- and T2DM-associated 1-deoxy-sphingolipids inhibits the migration of fibroblasts

**DOI:** 10.1016/j.jlr.2021.100122

**Published:** 2021-09-24

**Authors:** Gergely Karsai, Regula Steiner, Andres Kaech, Museer A. Lone, Arnold von Eckardstein, Thorsten Hornemann

**Affiliations:** 1 Institute of Clinical Chemistry, University Hospital Zürich, Zürich, Switzerland; 2 Center for Microscopy and Image Analysis, University of Zürich, Zürich, Switzerland

**Keywords:** sphingolipid, 1-deoxy-sphingolipids, metabolism, fumonisin B1, ceramide synthase, functional lipidomics, cell migration, ceramide, live cell imaging, T2DM, 1-deoxySA, 1-deoxy-sphinganine, 1-deoxySL, 1-deoxy-sphinglipid, 1-deoxySO, 1-deoxy-sphingosine, CerS, ceramide synthase, EA, Epon/Araldite, EM, electron microscopy, FADS3, fatty acid desaturase 3, FB1, fumonisin B1, Golgi, Golgi apparatus, HPF, high-pressure frozen, HSAN1, hereditary sensory neuropathy type 1, LCB, long-chain base, SA, sphinganine, SL, sphingolipid, SO, sphingosine, SPT, serine palmitoyltransferase, SPTLC1, serine palmitoyltransferase long-chain base subunit 1, S1P, sphingosine-1-phosphate, T2DM, type 2 diabetes mellitus

## Abstract

Hereditary sensory neuropathy type 1 (HSAN1) is a rare axonopathy, characterized by a progressive loss of sensation (pain, temperature, and vibration), neuropathic pain, and wound healing defects. HSAN1 is caused by several missense mutations in the serine palmitoyltransferase long-chain base subunit 1 and serine palmitoyltransferase long-chain base subunit 2 of the enzyme serine palmitoyltransferase—the key enzyme for the synthesis of sphingolipids. The mutations change the substrate specificity of serine palmitoyltransferase, which then forms an atypical class of 1-deoxy-sphinglipids (1-deoxySLs). Similarly, patients with type 2 diabetes mellitus also present with elevated 1-deoxySLs and a comparable clinical phenotype. The effect of 1-deoxySLs on neuronal cells was investigated in detail, but their impact on other cell types remains elusive. Here, we investigated the consequences of externally added 1-deoxySLs on the migration of fibroblasts in a scratch assay as a simplified cellular wound-healing model. We showed that 1-deoxy-sphinganine (1-deoxySA) inhibits the migration of NIH-3T3 fibroblasts in a dose- and time-dependent manner. This was not seen for a non-native, L-threo stereoisomer. Supplemented 1-deoxySA was metabolized to 1-deoxy-(dihydro)ceramide and downstream to 1-deoxy-sphingosine. Inhibiting downstream metabolism by blocking N-acylation rescued the migration phenotype. In contrast, adding 1-deoxy-sphingosine had a lesser effect on cell migration but caused the massive formation of intracellular vacuoles. Further experiments showed that the effect on cell migration was primarily mediated by 1-deoxy-dihydroceramides rather than by the free base or 1-deoxyceramides. Based on these findings, we suggest that limiting the N-acylation of 1-deoxySA could be a therapeutic approach to improve cell migration and wound healing in patients with HSAN1 and type 2 diabetes mellitus.

Sphingolipids (SLs) represent a structurally diverse class of lipids, which typically share the presence of a long-chain base (LCB) as a common structure (1). They are involved in essential cellular processes such as survival, proliferation, differentiation, metabolism, and apoptosis (2, 3, 4). SL metabolism is altered not only in a variety of monogenetic diseases (5) but also in several acquired conditions such as the metabolic syndrome, type 2 diabetes mellitus (T2DM), and cancer (6, 7, 8, 9, 10, 11, 12).

The enzyme serine palmitoyltransferase (SPT) catalyzes the first and rate-limiting step in the de novo synthesis of SLs, which is typically the condensation of palmitoyl-CoA and L-serine. The initially formed intermediate 3-keto-sphinganine is rapidly converted to sphinganine (SA). SA is then either N-acylated by ceramide synthases (CerS1-6) forming (dihydro)ceramides (13) or phosphorylated to SA-1-phosphate. While L-serine is the preferred substrate for SPT, the enzyme can also metabolize L-alanine and L-glycine to a certain extent. These alternative reactions form atypical 1-deoxy-sphingolipids (1-deoxySL) (14) that lack the C1 hydroxyl group of canonical SLs and are, therefore, neither converted to complex SLs nor phosphorylated. The lack of the phosphate group prevents their degradation by sphingosine-1-phosphate (S1P) lyase through the canonical catabolic pathway (14). However, like canonical SL, 1-deoxySL are N-acylated by CerS as well. The resulting 1-deoxy-dihydroceramides are metabolized by fatty acid desaturase 3 (FADS3) (15) and cytochrome P450 enzymes (16).

Several missense mutations in the genes encoding the serine palmitoyltransferase long-chain base subunit 1 (*SPTLC1*) and serine palmitoyltransferase long-chain base subunit 2 of SPT cause the hereditary sensory neuropathy type 1 (HSAN1) (17) by shifting the substrate preference of SPT from L-serine to L-alanine and thereby increasing the formation of 1-deoxySL (17, 18). HSAN1 is a peripheral axonal neuropathy, characterized by progressive sensory loss and neuropathic pain. In addition, HSAN1 patients suffer from wound healing defects and ulcers that frequently lead to osteomyelitis requiring amputations. These complications are rather specific features in HSAN1 and not commonly seen in other inherited neuropathies. Interestingly, patients with T2DM (19, 20, 21) show a similar clinical and metabolic phenotype with peripheral neuropathy, impaired wound healing, and elevated 1-deoxySLs although these patients have no mutations in the genes encoding the SPT subunits (12, 21, 22, 23).

The neurotoxicity of 1-deoxySLs has been recapitulated in cell culture (17, 24) and animal models (18, 25, 26, 27) by manipulating the exogenous supply of amino acid substrates. L-alanine supplementation increased 1-deoxySLs levels in a mouse model for HSAN1 and aggravated the neuropathy and skin phenotypes (25). In contrast, L-serine supplementation suppressed the formation of 1-deoxySL and improved neuropathy and skin robustness in both mice and humans with HSAN1 (25, 28, 29). This suggests, that 1-deoxySL also contribute to the skin pathology in HSAN1. However, their role in wound healing is still elusive.

Wound healing is a highly coordinated process that involves the orchestrated migration of several cell types into the traumatic area. First, immune cells invade into the lesion, followed by fibroblasts and keratinocytes from the periphery. This process is followed by cell proliferation, the synthesis and release of extra cellular matrix components, and finally the reinnervation and angiogenesis to supply the growing skin tissue with oxygen and nutrients (30). All these cellular processes require highly coordinated changes in the cytoskeletal dynamics. 1-DeoxySLs interfere with stress fiber formation and cytoskeletal dynamics in yeast (31), worms (32), and mammalian cells (17, 22, 33, 34, 35). A cytostatic effect of 1-deoxySLs has also been demonstrated in various cancer cell models (11, 36, 37, 38, 39, 40). In addition, 1-deoxySLs have been implicated in hypoxia-induced tissue injury and the formation of aggregated actin (35). Based on these reports, we investigated the hypothesis that 1-deoxySLs interfere with cell migration as a possible explanation for the impaired wound healing process that is associated with elevated 1-deoxySL.

## Materials and methods

### Cell culture

NIH-3T3 cells were obtained from the ATCC and cultured in DMEM (Thermo Fisher Scientific) supplemented with 10% fetal calf serum and 1% penicillin/streptomycin at 37°C in 5% CO_2_ atmosphere.

### SPTLC1 mutant generation

SPTLC1 cDNA was amplified from the pCDNA3.1-SPTLC1-V5 construct (41), with primers SPTLC1 Clover_F: 5′-CGCGGATCCATGGCGACCGCCACGGAGCAG-3′ and SPTLC1 Clover_R: 5′-CCGGAATTCGAGCAGGACGGCCTGGGCTAC-3′. The amplicon was cloned upstream of the improved green fluorescent protein (Clover) (42) using BamHI and EcoRI in the pCDNA3-Clover vector (Addgene #40259). Constructs were identified by Sanger sequencing and Western blot for expression. The plasmid vector containing SPTLC1-Clover was used to generate SPTLC1-C133W-Clover point mutant using site-directed mutagenesis with HPLC-grade purified primers, SPTLC1_C133W_fw: 5′-ggggacccagaggattttatggcacatttgatgttc-3′ and SPTLC1_C133W_rv: 5′-aggtacccacgccatacttctttagagatgctaaagc-3′ and high-fidelity Phusion DNA polymerase (Thermo Fisher Scientific).

### SPTLC1 mutant cell line generation

NIH-3T3 fibroblasts were transfected with Lipofectamine 3000 (Thermo Fisher Scientific) according to the supplier protocol with the WT or mutant SPTLC1-EGFP construct, and transfected cells were kept under 1,000 μg/ml G418 (Thermo Fisher Scientific) selection in DMEM for four passages. Single-cell colonies were raised from EGFP-positive cells after fluorescence-activated cell sorting.

### Scratch assay (live-cell imaging)

NIH-3T3 cells (50 000/well) were seeded in a 48-well plate 48 h before the assay. To prevent cell proliferation during the assay, cells were treated with 10 μg/ml Mitomycin C (Sigma-Aldrich) for 2 h, then a scratch was introduced in the middle of the well by a 20 μl pipette tip. The well was rinsed once with DMEM and replaced with DMEM supplemented with the corresponding treatment (lipids were acquired from Avanti Polar Lipids or produced internally (43)). Then, the plate was transferred to the live-cell imaging microscope (Olympus IX81) fitted with an incubator (humidified atmosphere, 37°C, 5% CO_2_) and a motorized stage. Phase-contrast images were taken at 6.4× magnification every 30 min for 48 h, with the Hamamatsu (C11440) detector at 1 mega pixel (1,024 × 1,024 pixel) 16 bit.

### Live/dead stain

After the scratch assay (see above), cells were rinsed with PBS and stained with 3 μmol/l calcein-acetoxymethyl (Thermo Fisher Scientific) and 2.5 μmol/l propidium iodide (Thermo Fisher Scientific) dissolved in 1× PBS for 15 min and rinsed in PBS. Images were acquired in PBS on a Zeiss Axiovert 200M with a Plan-Apochromat 20 ×/0.8 and Hamamatsu ORCA-ER EMCCD camera (C4742) with the appropriate filters.

### Lipidomics

Lipid extraction was performed as described previously (44). Shortly, 0.5–2.5 million cells were suspended in 20 μl PBS, and 1 ml of a mixture of methanol:methyl tert-butyl ether:chloroform 4:3:3 (v/v/v) was added. The methanol:methyl tert-butyl ether:chloroform mix was fortified with 100 pmol/ml of the internal standards: d7-sphinganine (d18:0), d7-sphingosine (d18:1), dihydroceramide (d18:0:12:0), 1-deoxy-dihydroceramide (m18:0/12:0), ceramide (d18:1/12:0), 1-deoxy-ceramide (m18:1/12:0), glucosylceramide (d18:1/8:0), sphingomyelin (18:1/12:0), and 50 pmoles/ml d7-S1P. After brief vortexing, the samples were continuously mixed in a Thermomixer (Eppendorf) at 37°C (1,400 21 rpm, 20 min). Protein precipitation was obtained after centrifugation for 5 min, 16,000 *g*, 25°C. The single-phase supernatant was collected, dried under N_2_, and stored at –20°C until analysis. Before analysis, the dried lipids were dissolved in 100 μl methanol. Liquid chromatography was done according to (45), with some modifications. The lipids were separated using a C30 Accucore LC column (Thermo Fisher Scientific, 150 mm × 2.1 mm × 2.6 μm) using the following mobile phases: (A) acetonitrile:water (2:8) with 10 mM ammonium acetate and 0.1% formic acid, (B) isopropanol: acetonitrile (9:1) with 10 mM ammonium acetate, and 0.1% formic acid, and (C) methanol at a flow rate of 0.3 ml/min. The following gradient was applied: (1) 0.0–1.5 min (isocratic 70% A, 20% B, and 10% C), (2) 1.5–18.5 min (ramp 20%–100% B), (3) 18.5–25.5 min (isocratic 100% B), and (4) 25.5–30.5 min (isocratic 70% A, 20% B, and 10% C). The liquid chromatography was coupled to a hybrid quadrupole-orbitrap mass spectrometer Q-Exactive (Thermo Fisher Scientific, Reinach, BL, Switzerland), samples were analyzed in the positive mode using a heated electrospray ionization interface. The following parameters were used: spray voltage 3.5 kV, vaporizer temperature of 300°C, sheath gas pressure of 20 AU, aux gas of 8 AU, and capillary temperature of 320°C. The detector was set to an MS2 method using a data-dependent acquisition with Top10 approach with stepped collision energy between 25 and 30. A 140,000 resolution was used for the full spectrum and a 17,500 for MS2. A dynamic exclusion filter was applied, which will exclude fragmentation of the same ions for 20 s. Identification and quantification was achieved as previously published (15, 44), with the following identification criteria:

1) Resolution with an accracy of 5 ppm from the predicted mass at a resolving power of 140,000 at 200 m/z.
2) Isotopic pattern fitting to expected isotopic distribution.
3) Matching retention time to in-house lipid databes or synthetic standards if available.
4) Specific fragmentation patterns:
  a) Free sphingoid base: [M+H]^+^ → [M+H − H_2_O]^+^ and [M+H − H_2_O − H_2_O]^+^;
  b) 1-Deoxy-sphingoid base: [M+H]^+^ → [M+H − H_2_O]^+^;
  c) S1P: [M+H]^+^ → [M+H − H_2_O]^+^ and [M+H − H_2_O − HPO_4_]^+^;
  d) (1-Deoxy)ceramide: [M+H]^+^ → [M+H − H_2_O]^+^ and [M+H − H_2_O − (fatty acid)]^+^;
  e) Hexosylceramide: [M+H]^+^ → [M+H − H_2_O]^+^ and [M+H - H_2_O − (fatty acid) − glycoside]^+^;
  f) Sphingomyelin: [M+H]^+^ → [M+H − H_2_O]^+^ and [M+H − H_2_O − (fatty acid) − PO_4_ choline]^+^.

Data analysis was performed using TraceFinder 4.1 (Thermo Fisher Scientific) for peak picking, annotation, and matching to an in-house lipid database. Quantification was done using single-point calibration. Pooled samples at 5 concentrations were used as quality controls.

### Metabolic labeling

Cells (250,000) were seeded in 2 ml fresh medium in 6-well plates (BD Falcon) and cultured for 2 days, reaching approximately 70%–80% confluence. The medium was exchanged for L-serine- and L-alanine-free DMEM (Genaxxon Bioscience), containing 10% FBS (Thermo Fisher Scientific; FSA15043) and 1% penicillin and streptomycin (100 units/ml and 0.1 mg/ml, respectively; Millipore Sigma-Aldrich). Two hours after medium exchange, isotope-labeled (2,3,3)-d3-^15^N-L-serine (1 mM) and (2,3,3,3)-d4-L-alanine (2 mM) was added (Cambridge Isotope Laboratories).

### Plasma samples

HSAN1 plasma was obtained with written informed consent from a previously published study (46).

### Vacuole size distribution

The diameter of the vacuoles was measured after 16 hours of 2 μM sphingosine (SO) or 1-deoxy-sphingosine (1-deoxySO)^Δ14Z^ treatment with Fiji's (47) measure tool on phase-contrast images acquired by confocal laser scanning microscopy (Leica SP8).

### Visualization of macropinocytosis

NIH-3T3 cells grown for 24 h on 12-mm coverslips and treated according to experimental procedure in the presence of 0.5 mg/ml Dextran-488 (10,000 MW, Thermo Fisher Scientific). Cells were fixed with 4% paraformaldehyde for 30 min, rinsed three times in PBS, and mounted on glass microscopic slides with ProLong Diamond Antifade Mountant (Thermo Fisher Scientific).

### Lipid droplet staining

NIH-3T3 cells grown for 24 h on 12-mm coverslips and treated according to experimental procedure were fixed with 4% paraformaldehyde for 30 min and washed in PBS. Cells were incubated with 0.2 μg/ml BODIPY 493/503 (Thermo Fisher Scientific) in PBS for 1 h, washed again, and mounted on glass microscopic slides with ProLong Diamond Antifade Mountant (Thermo Fisher Scientific).

### Fluorescent immunohistochemistry

NIH-3T3 cells grown on 12-mm coverslips and treated according to the experimental procedure were fixed with 4% paraformaldehyde for 30 min and washed in PBS. The blocking buffer (1× PBS, 5% BSA, 1% NGS, and 0.25% Triton X-100, Sigma-Aldrich) was added to the cells for 2 h, followed by overnight incubation with the primary antibodies at 4°C. To visualize the ER, anti-calnexin antibody (Sigma-Aldrich C4731, 1:2,000), for mitochondria, anti-AIF antibody (Thermo Fisher Scientific 4E7E11, 1:2,000), and for the Golgi apparatus (Golgi), anti-Golgin-97 antibody (Thermo Fisher Scientific #PA5-30048, 1:2,000) were diluted in the blocking buffer. Subsequently, coverslips were thoroughly washed with PBS and incubated with secondary antibodies (Jackson ImmunoResearch) diluted 1:1,000 in the blocking solution for 4 h and thoroughly washed again and mounted on glass microscopic slides with ProLong Diamond Antifade Mountant (Thermo Fisher Scientific). Confocal stacks were acquired on a confocal laser scanning microscope (Leica SP8; Leica Microsystems, Wetzlar, Germany), with a 63× objective (HC PL APO CS2 63× N.A. 1.4 oil) at 90.4 nm × 90.4 nm × 300 nm (x × y × z) resolution. Images were analyzed using Fiji image processing package (47).

### Electron microscopy

#### High-pressure freezing

NIH-3T3 cells were grown for 24 h on carbon-coated sapphire discs and treated according to experimental procedures. The sapphire discs were carefully sandwiched with a 1-hexadecene (Sigma-Aldrich) wetted aluminum specimen carrier with an indentation of 100 μm and a spacer ring, and immediately high pressure–frozen (HPF) using an automated Leica EM HPM100 high-pressure freezing machine (Leica Microsystems, Austria). Samples were stored in liquid nitrogen until further processing.

#### Freeze substitution fixation for EM

HPF discs containing cell monolayers were transferred to 2 ml safe-lock Eppendorf tubes containing anhydrous acetone with 1% OsO_4_ at −90°C. Substitution was performed in an automated substitution machine (Leica EM AFS) at −90°C for 7 h, −60°C for 6 h, −30°C for 5 h, and 0°C for 1 h with transition gradients of 30°C per hour.

#### Embedding and preparation for transmission electron microscopy and image acquisition

Samples in anhydrous acetone were embedded in Epon/Araldite (EA) essentially as described by Hohenberg *et al.* (48, 49), by incubating the samples in 66% EA in acetone for 8 h before transfer in 100% EA and polymerization at 60°C for 40 h.

Ultrathin cross-sections of cells of 50 nm were cut with a 45° diamond knife (Diatome) using an ultramicrotome (Reichert) and put on Formvar-coated single-slot grids (Ted Pella Inc).

Images were acquired with a Philips CM100 or a FEI Tecnai G2 Spirit transmission electron microscope (FEI, Eindhoven, The Netherlands) at an acceleration voltage of 80 kV or 120 kV using a Gatan Orius 1000 camera (Gatan Inc).

## Results

### Automated scratch assay analysis

**Fig. 1:**
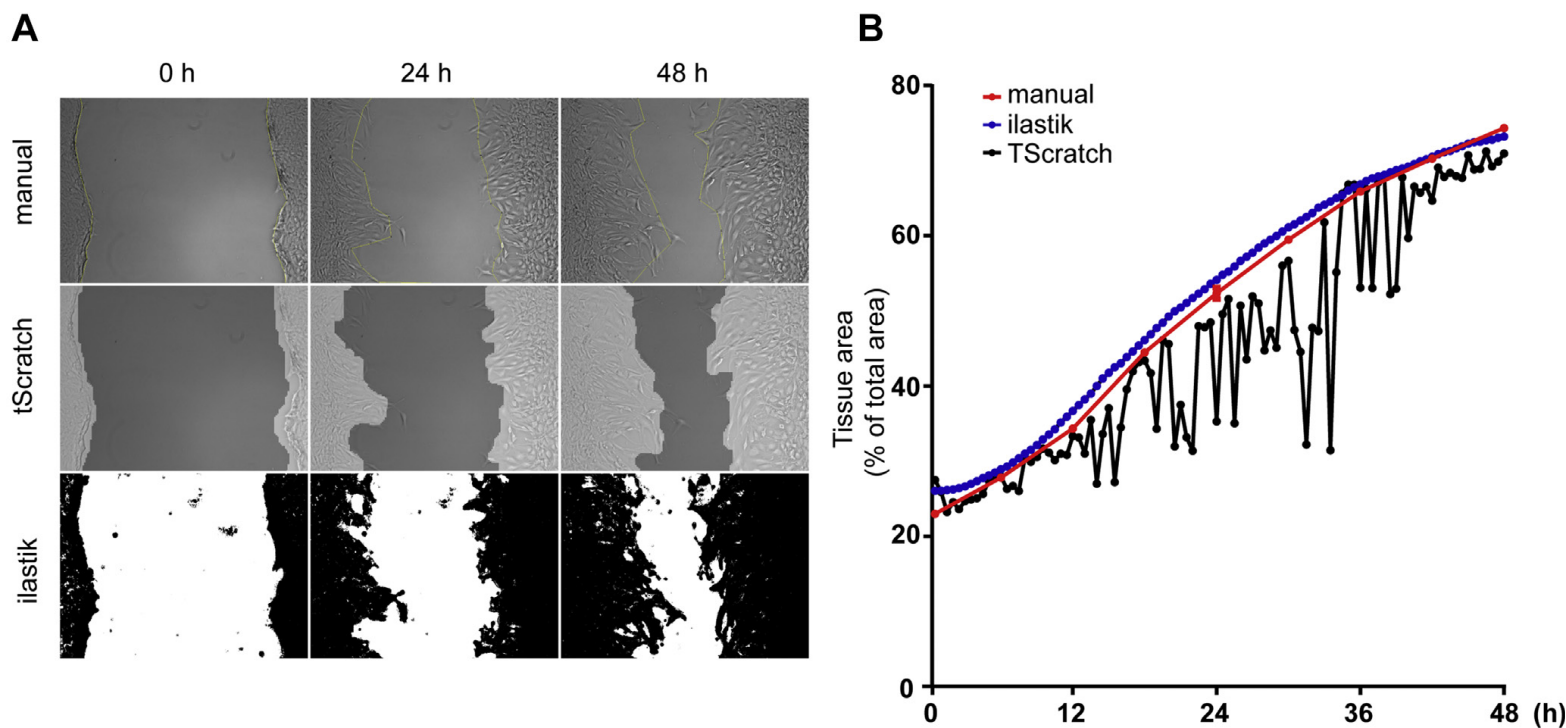
Automated scratch assay analysis. A: Representative pictures of the scratch assay that were analyzed manually, with TScratch and ilastik. Scale bar, 100 μm. B: Comparison between manual (three times) and the two automated segmentation methods over time. Manual (red, ±SD) segmentation and automated segmentation by ilastik (blue) are consistent and comparable, whereas automated segmentation by TScratch (black) was inconsistent over time.

To investigate whether 1-deoxySLs affect cell migration, we developed a scratch assay protocol to analyze the migration of NIH-3T3 cells over time. After cells were grown to confluency, a pipette tip was used to introduce a cell-free gap in the middle of the well (Fig. 1A). Cell migration was recorded for 48 h by live-cell imaging. Recorded images were analyzed by Ilastik, a freely available interactive picture segmentation software (50). After an initial training phase, the machine-learning algorithm was able to differentiate between tissue and gap area automatically (Fig. 1A). The obtained results were more robust than those from TScratch, another free software tool for the analysis of migration assays (51), and consistent with manual quantification (Fig. 1A, B). As the manual scratch can vary in its initial gap size, we calculated the relative change in the tissue area from baseline over time (% change of the total area) (supplemental Fig. S1A). Within the experimental timeframe of 48 h, the cells did not close the gap fully and therefore remained in a migratory phase for the whole experiment (supplemental Fig. S1B). Cell migration was independent of the initial gap size.

### 1-DeoxySLs inhibit migration of NIH-3T3 cells in culture

**Fig. 2:**
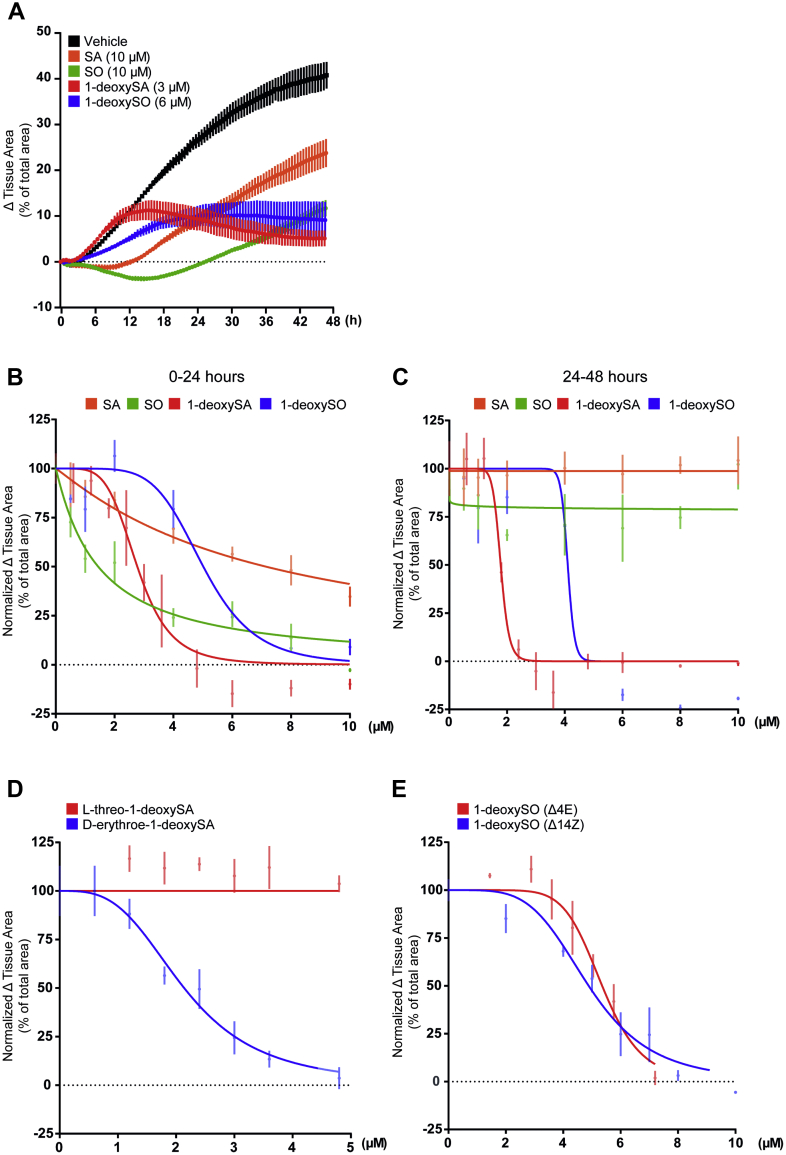
1-DeoxySLs inhibit the migration of NIH-3T3 fibroblasts in culture. Cell migration was monitored for up to 48 h in the presence of sphinganine (SA), sphingosine (SO), 1-deoxy-sphinganine (1-deoxySA), or 1-deoxy-sphingosine (1-deoxySO^Δ14Z^). A: Characteristic migration profiles of NIH3T3 fibroblasts treated with vehicle (black) exhibit a hyperbolic migration curve, whereas 1-deoxySL-treated cells continue to migrate for 12–16 h before migration stops completely after 24 h. In contrast, SA- and SO-treated cells showed an initial delay in migration for 12–18 h, followed by linear migration profile between 24 and 48 h. B: Dose-response curves of the initial 24 h after adding the lipids. Initially, SA (yellow) and SO (green) showed linear dose responses, while the dose responses to 1-deoxySA (red) and 1-deoxySO^Δ14Z^ (blue) were sigmoidal. C: From 24 to 48 h, SA-treated (yellow) and SO-treated (green) cells showed concentration-independent and constant migration rates, whereas the addition of 1-deoxySA (red) and 1-deoxySO^Δ14Z^ (blue) showed similar sigmoidal dose responses as seen during the first 0–24 h. D: Dose response of the two 1-deoxySA stereoisomers D-erythro and L-threo. Only the native D-erythro form (blue) affected migration, whereas the L-threo isoform (red) had no effect in the tested concentrations. E: Dose response curves for natural (Δ14Z) and synthetic (Δ4E) 1-deoxySO isomers. Both forms had similar effect on the migration. Error bars indicate the mean ± SEM. Data are representative of at least three independent experiments. The curves represent nonlinear fitting using GraphPad Prism 8.

Supplementing NIH-3T3 cells with increasing concentrations of 1-deoxySA or 1-deoxySO^Δ14Z^ reduced cell migration in a dose-dependent manner (Fig. 2A–C), without compromising cell viability (supplemental Fig. S2A). The inhibition was more pronounced for 1-deoxySA (IC_50_ 2.6 μM) than for 1-deoxySO^Δ14Z^ (IC_50_ 4.8 μM) (Fig. 2A–C). To test whether the inhibition is specific, we compared the effect of native D-*erythro*-1-deoxySA with the non-native, synthetic L-*threo* stereoisomer of 1-deoxySA. Only the native D-*erythro* but not the synthetic L-*threo* isomer affected cell migration (Fig. 2D). In contrast, supplementing SA or SO caused an initial (first 24 h of the assay) dose dependent but transient rounding of the cells associated with a delay in migration that was more pronounced for SO than for SA (Fig. 2A, B) and was not observed for 1-deoxyLCBs (supplemental Videos S2–S4). After this initial lag phase, the cells migrated with a constant rate, which was not further affected by the added LCBs (Fig. 2C).

Canonical SLs contain a Δ4E double bond, whereas 1-deoxySO and the 1-deoxyCer bear an atypical Δ14Z double bond (15, 43). To test whether the conformation or position of the double bond has an influence on migration, we compared 1-deoxySO (Δ14Z) with a synthetic 1-deoxySO (Δ4E) stereoisomer. Both compounds had a comparable inhibitory effect on the migration (Fig. 2E), indicating that the type or the position of the double bond is not relevant for the inhibition.

### CerS inhibition by FB1 rescued the migration effect in NIH-3T3 cells

**Fig. 3:**
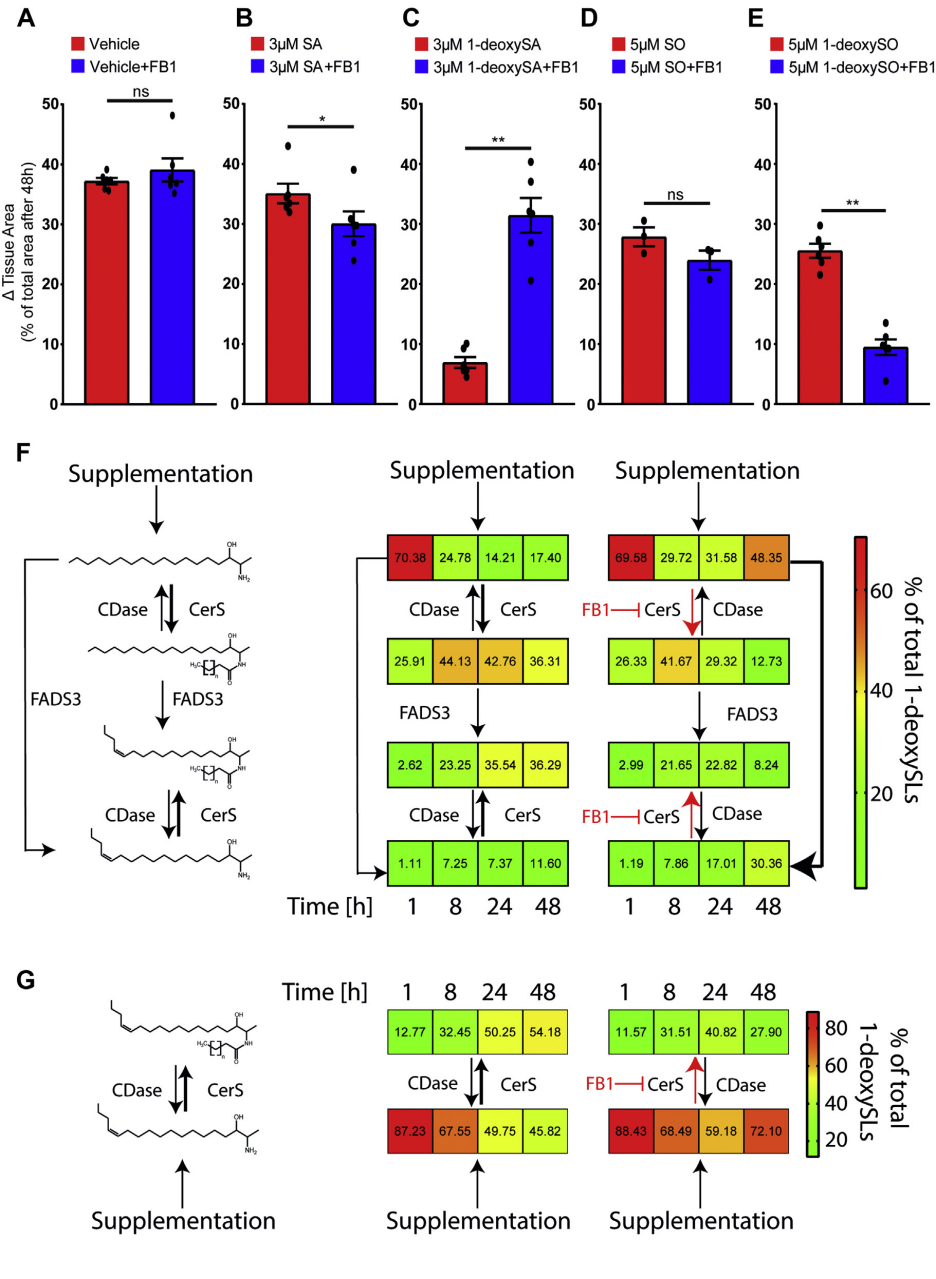
CerS inhibition by FB1 rescues the 1-deoxySA-caused migration effect. Migration of NIH3T3 fibroblasts supplemented with LCBs ± FB1 for 48 h (A) FB1 (35 μM) alone had no effect on migration (B) but showed a mild inhibitory effect on the migration of SA-supplemented cells. C: FB1 (7 μM) reversed the inhibitory effect in 1-deoxySA-treated cells. D: FB1 (35 μM) in combination with SO did not reduce migration. E: FB1 (35 μM) in combination with 1-deoxySO^Δ14Z^ inhibited cell migration even further. Error bars indicate the mean ± SEM. Data are representative of at least three independent experiments. ∗*P* < 0.05 and ∗∗*P* < 0.01. F and G: Time dependent changes in the 1-deoxy(dh)Cer profile in NIH-3T3 fibroblasts treated with (isotope labeled) 1-deoxySA (3 μM) or 1-deoxySO^Δ14Z^ either in the presence or absence of FB1 (7 μM). The data (values in the box) represent the mean of 1-deoxySL class, which was normalized to the total 1-deoxySLs; the coloring of the boxes represents the abundance of individual values. Data are representative of at least three independent experiments. CerS, ceramide synthase; 1-deoxySA, 1-deoxy-sphinganine; 1-deoxySL, 1-deoxy-sphinglipid; FB1, fumonisin B1; LCB, long-chain base; ns, not significant; SL, sphingolipid.

With an IC_50_ of 2.6 μM, 1-deoxySA was the more potent inhibitor than 1-deoxySO^Δ14Z^ (IC_50_ 4.8 μM). This suggests that either the free LCB (1-deoxySA) or its downstream product 1-deoxy-dihydroceramide (1-deoxydhCer) is relevant for the reduced migration. To distinguish between the two possibilities, we inhibited the N-acylation of the (1-deoxy)LCBs with fumonisin B1 (FB1), a pan CerS1-6 inhibitor. Alone or in combination with SA or SO, FB1 had a negligible impact on migration (Fig. 3A, B, D). However, the inhibitory effect of 1-deoxySA was reversed in the presence of FB1 (Fig. 3C). Surprisingly, 1-deoxySO^Δ14Z^ in combination with FB1 did not rescue but exacerbated the inhibitory effect (Fig. 3E).

As 1-deoxySA and 1-deoxySO^Δ14Z^ in combination with FB1 showed diverging results, we investigated whether FB1 had the same inhibitory effect on the metabolism of the two LCBs. The supplemented 1-deoxySA was rapidly taken up by cells and N-acylated to 1-deoxydhCer, which were gradually desaturated to 1-deoxyCer (Fig. 3F); 1-deoxySO was either deliberated from 1-deoxy-Cers by ceramidase activity or resulted from direct desaturation of 1-deoxySA by FADS3 (15). The supplemented 1-deoxySO^Δ14Z^ was also rapidly taken up by cells and N-acylated to 1-deoxyCer (Fig. 3G). Treatment with FB1 resulted in reduced N-acylated base/free base ratio for 1-deoxySA as well as for 1-deoxySO^Δ14Z^, with a similar tendency (supplemental Fig. S2B, C). In both cases, FB1 showed a delayed response (Fig. 3F, G, supplemental Fig. S2B, C) to inhibit the N-acylation of the supplemented 1-deoxyLCBs.

We also analyzed the changes in the N-acyl profile over time in the presence and absence of FB1. 1-DeoxySA was predominantly conjugated with palmitate (C16:0) by CerS5/6 in 1-deoxydhCer form, whereas in 1-deoxyCer form, the dominant species were conjugated with very-long-chain fatty acids (C22:0, 24:0, 24:1) by CerS2 (52). Inhibitions of CerSs by FB1 first reduced the levels of 1-deoxydhCer with long-chain FAs (C16:0), whereas the effect on very-long-chain FAs (C22:0, 24:0, 24:1) was delayed and less prominent (supplemental Fig. S3A). In 1-deoxySO^Δ14Z^-supplemented cells, CerS2 products dominated the 1-deoxyCer profile. FB1 also preferentially decreased the CerS5/6 products, as was seen with 1-deoxySA (supplemental Fig. S3B).

### 1-DeoxySO^Δ14Z^ but not 1-deoxySA induces vacuole formation in NIH-3T3 cells

**Fig. 4:**
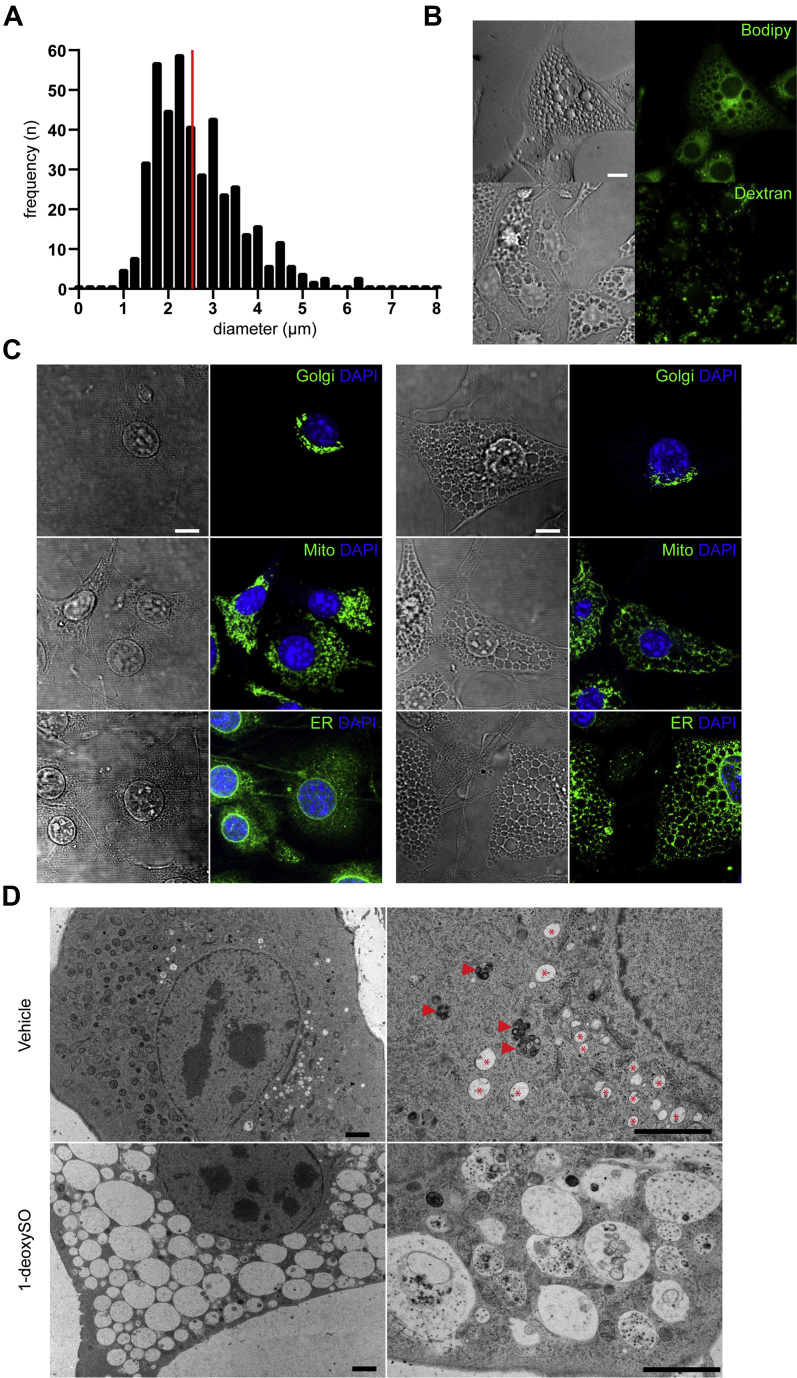
1-DeoxySO^Δ14Z^ treatment induces vacuole formation in NIH-3T3 fibroblasts. Fibroblasts were supplemented with 1-deoxySO^Δ14Z^ (2 μM) for 16 h. A: The average size of vacuole diameter distribution after 1-deoxySO^Δ14Z^ treatment, upon manual assessment, is 2.54 μm ±0.47 (red line). Data represent the average of 436 vacuoles from 11 cells ± SEM. B: In addition to 1-deoxySO^Δ14Z^, cells were incubated with either BODIPY or Dextran. The vacuoles were stained neither with BODIPY nor with Dextran. C: Cells were exposed to SO (2 μM, left) or 1-deoxySO^Δ14Z^ (2 μM, right) for 16 h and stained for the Golgi, the mitochondria, and the ER using specific organelle markers. The ER and the mitochondria appeared to be surrounded by the vacuoles. Golgi morphology was not altered. Scale bars, 10 μm. D: Electron microscopic analysis of cells supplemented with 1-deoxySO^Δ14Z^ (2 μM) or vehicle (ethanol) for 16 h. The cytoplasm of 1-deoxySO^Δ14Z^-treated cells showed tightly packed vacuoles of which the larger ones appear to be empty, whereas the small ones appear to contain cell debris. The ER and the mitochondria are squeezed between the vacuoles, lysosomes (red star), and multivesicular bodies (red arrow head). Scale bar, 2 μm. Data are representative of at least three independent experiments. 1-deoxySO, 1-deoxy-sphingosine; Golgi, Golgi apparatus.

Besides the migration effect, we observed a massive appearance of large intracellular vacuoles in 1-deoxySO^Δ14Z^-supplemented cells (Fig. 4A, B); below the 1-deoxySO IC_50_ value (4.8 μM), these vacuoles disappeared over time (supplemental Video S3); however, at a higher concentration, they persisted, which coincides with cessation of migration (supplemental Video S4). The vacuoles were only detected in cells treated with 1-deoxySO^Δ14Z^ but not in cells treated with SA, SO (Fig. 4C), or 1-deoxySA (supplemental Videos S1, S2, S5, and S6). The average diameter of the vacuoles was 2.54 ± 0.47 μm SEM (Fig. 4A) and labeled negative for BODIPY and Dextran (Fig. 4B). Using organelle-specific markers, we tested whether the morphology of the Golgi, the mitochondria, or the ER was altered in response to the vacuole formation. For the Golgi, no obvious effect was observed, whereas the shape of ER and mitochondria seemed to be altered and tightly surrounded by the vacuoles (Fig. 4C). However, subsequent electron microscopy (EM) revealed that mitochondria and ER were not directly associated with the vacuoles but rather squeezed between them in the remaining cytoplasmic space (Fig. 4D). The vacuoles were much larger than multivesicular bodies (red arrow-head) or lysosomes (red star). From the EM analysis, it also appeared that the small vacuoles contained some cell debris, whereas the larger structures appeared empty.

## Discussion

The association of elevated 1-deoxySL with peripheral neuropathy is well established and confirmed in cell culture (17, 24), animal models (18, 25, 26, 27), and clinical studies (28, 29). Conditions where 1-deoxySL are elevated (e.g., HSAN1 and T2DM) are often associated with slow-healing wounds and ulcers. Wound healing requires a well-orchestrated series of events that involves migration, proliferation, and differentiations of various cell types. These processes rely, to a great extent, on the dynamic and functional rearrangements of the cytoskeleton (see for review (53)). Because 1-deoxySLs were shown to interfere with cytoskeleton dynamics in yeast (31), worm (32), and mammalian cells (17, 22, 33, 34, 35) and also to inhibit cell growth (11, 54), we were interested to test whether 1-deoxySLs also have an effect on cell migration—one of the key processes in wound healing.

Cell migration was analyzed by a protocol that combines the scratch assay with automated, quantitative, high-throughput image analysis. This live-cell imaging approach allowed performing the experiments in a multiwell format with high temporal and spatial resolution. Image analysis was done with the freely available image classification tool Ilastik (50). This automatic workflow, allowed a high throughput analysis of cell migration with high accuracy and excluding the risk of bias that is often a problem with manual quantifications.

It is currently not clear whether the intracellular biosynthesis of 1-deoxySL or their uptake from the circulation is responsible for the toxic effects. In HSAN1, where the mutant SPT is systemically expressed, 1-deoxySLs are elevated in both tissue and blood, whereas in T2DM, levels are primarily elevated in the blood. As 1-deoxySL are transported by LDL and VLDL (19), the cellular uptake could be further modulated by the expression of lipoprotein receptors such as the LDL or scavenger receptors. We therefore decided to use an ectopic supplementation model to analyze the effect of 1-deoxySL on cell migration. We showed that ectopically added 1-deoxySA and 1-deoxySO^Δ14Z^ inhibited the migration of NIH-3T3 fibroblasts in a dose- and time-dependent manner. The inhibition was more potent for 1-deoxySA (IC_50_ 2.6 μM) than for 1-deoxySO^Δ14Z^ (IC_50_ 4.8 μM) and in a similar concentration range as we find it in plasma of individuals with HSAN1 (0.6–2.5 μM) (55) or T2DM (0.3–0.9 μM) (21). The effect was only seen for the natural D-*erythro* form but not for the synthetic L-*threo* stereoisomer. Lipid profiling showed that both 1-deoxySA stereoisomers were resorbed similarly (supplemental Fig. S5A) but metabolized with different efficacy. The D-erythro form was metabolized to 1-deoxydhCer, 1-deoxyCer, and 1-deoxySO, whereas the L-threo form was mostly converted to 1-deoxydhCer although about one-third also remained as a free LCB (supplemental Fig. S5B). This argues for a specific mechanism that is selective to head group modifications and desaturation and excludes a general toxic effect of the LCB itself.

Supplementation with canonical LCBs (SA and SO) resulted in an initial but transient cell rounding and migration block but had no further effect on migration at later time points. The initial rounding and lag phase had been described previously for SO (56) but was not observed for 1-deoxySLs. A possible explanation could be a transient raise in S1P levels that is quickly formed from the supplemented SO (data not shown). It is known that S1P affects cell migration via S1P receptors 1 and 2 (57), which makes it difficult to distinguish between the SO- and S1P-mediated effects. However, as 1-deoxySL cannot be phosphorylated, a change in S1P cannot explain their effect on migration. Canonical SLs have a Δ4E DB that is introduced by DEGS1, whereas 1-deoxySLs have a Δ14Z DB that is introduced by FADS3 (15, 43). We tested whether the double bond positions have an impact on migration. Both the native 1-deoxySO^Δ14Z^ and the non-native synthetic stereoisomer 1-deoxySO^Δ4E^ had a similar effect and were less potent than 1-deoxySA, indicating that the presence, but not the double bond position, is of relevance.

FB1 inhibits CerSs and therefore prevents the N-acylation and downstream metabolism of LCBs. The observed protective effect of FB1 is in line with other reports showing that FB1 generally reduces the toxicity of 1-deoxySA (22, 34, 58). Surprisingly, the migration phenotype of 1-deoxySO^Δ14Z^ was exacerbated in presence of FB1. Both 1-deoxySA and 1-deoxySO^Δ14Z^ are N-acylated by CerS forming 1-deoxydhCer and/or 1-deoxyCer species, respectively. The fact that FB1 enhanced the effect of 1-deoxySO^Δ14Z^ by blocking its N-acylation could indicate that 1-deoxySO^Δ14Z^ itself could be a potent inhibitor. However, physiologically, this is likely of less relevance, as 1-deoxySO^Δ14Z^ as a free LCB is normally only present in little amounts.

We also tested whether supplementing long-chain 1-deoxydhCer (m18:0/24:1 and d18:0/24:0) had any effect on migration. The addition of these lipids showed no effect (supplemental Fig. S4A), but subsequent lipid analysis revealed that both metabolites are not resorbed efficiently by the cells (supplemental Fig. S4B). This agrees with earlier reports that showed that the length of the N-acyl chain correlates inversely with the uptake of the lipid (59, 60).

To our surprise and not directly related to the migration effect, we observed a massive vacuole formation in cells treated with 1-deoxySO^Δ14Z^. The vacuoles varied in sizes (1–6 μm) and were not formed in the presence of 1-deoxySA. Vacuole formation in response to 1-deoxySL treatment had been reported earlier (61), but the previously reported vacuoles were positive for BODIPY staining. However, the here-formed vacuoles were not sensitive to BODIPY, indicating that they are not made of neutral lipids. In light microscopy, the vacuoles seemed to be associated with the ER and the mitochondria, but subsequent EM revealed that these organelles were actually squeezed into the void volume of the cytoplasmic space rather than being directly associated with the vacuoles. In EM, the vacuoles showed a great heterogeneity in size (1–6 μm, mean 2.54 μm) and it appeared that the smaller structures contained cell debris, whereas the larger vacuoles seemed to be empty. Similar vacuoles were described for methuosis (62)—a recently described nonapoptotic cell death pathway. The hallmark for this form of cell death is a displacement of the cytoplasm by large fluid-filled vacuoles derived from macropinosomes. Macropinosomes can engulf large amounts extracellular fluid, which could be a response of the cells to the supplemented 1-deoxySO^Δ14Z^. However, the 1-deoxySO^Δ14Z^-related vacuoles were negative for Dextran staining, which is a marker for macropinosome formation and methuosis. Alternatively, the vacuoles could be related to a disturbed endosomal trafficking that has been reported previously in cells treated with SL analogs (63, 64). Recently, analogs of 1-deoxySO^Δ4E^ were identified as potent acid ceramidase (ACER3) inhibitors (65), which could induce lysosomal swelling; however, their effect on cell morphology was not investigated. Although potentially relevant for 1-deoxySL-mediated toxicity, the nature of these vesicles is not yet clear and needs to be addressed in more detail in future work.

The study presented here has some limitations. The in vitro scratch assay used in this study is a widely accepted tool to study wound healing; however, it cannot fully recapitulate the complexity of in vivo wound healing. Plasma 1-deoxySLs are in LDL and VLDL particles, predominantly in N-acylated form representing the liver CerS expression profile. However, in our assay, we used 1-deoxyLCBs; thus, their N-acylation is determined by the CerS expression profile of the host cell and it might be different from that of the liver. However, the 1-deoxySL profile of HSAN1 cells or plasma is comparable with that of 1-deoxyLCB-supplemented cells (supplemental Fig. S6). In addition, we investigated the acute effect (up to 48 h) of ectopically supplemented 1-deoxyLCBs, whereas patients with elevated 1-deoxySLs levels develop wound-healing defects over a period of several years (or decades). To investigate the impact on migration, and not the combination of migration and cell proliferation, we treated cells with Mitomycin C, a chemotherapeutic agent, which further limits the complexity of our model system and potentially affects cell response to further treatments.

In summary, we showed that the presence of 1-deoxySA and to a lower extent 1-deoxySO^Δ14Z^ reduces cell migration in vitro. The addition of FB1 reversed the effect, indicating that the relevant metabolite is formed downstream of 1-deoxySA. There is increasing evidence that ceramides with certain N-acyls have distinct effects in cells (10). However, to which extent the type and length of the N-acyl chain contributes to this effect is not yet clear and needs further investigation.

## Data availability

The data supporting this study are available in the article and are available from the corresponding author upon reasonable request.

## Supplemental data

This article contains supplemental data.

## Conflict of interest

The authors declare that they have no conflicts of interest with the contents of this article.
