# Supplementary material for: Metabolism of HSAN1- and T2DM-associated 1-deoxy-sphingolipids inhibits the migration of fibroblasts
Source: J Lipid Res. 2021 Sep 24;62:100122. doi: 10.1016/j.jlr.2021.100122 (PMC8521209; doi:10.1016/j.jlr.2021.100122)
Supplement: Supplemental Figs. S1–S6 [file mmc1.docx]

**Supplementary Information**


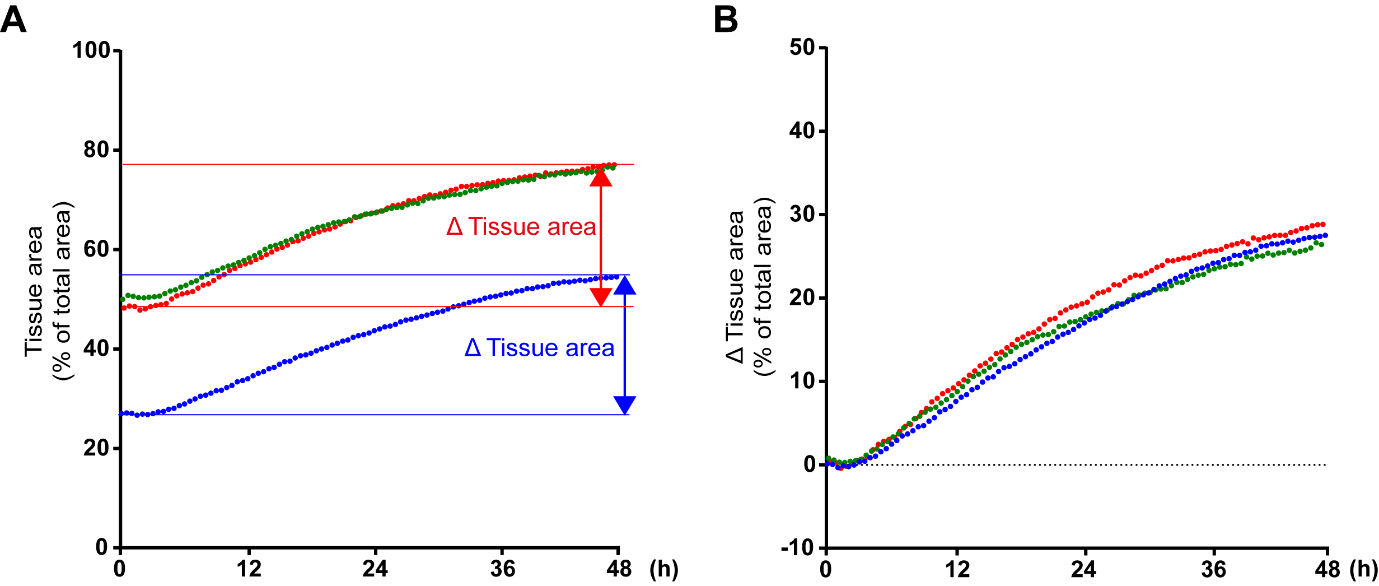


**Supplementary Figure 1.** **(A)** The variability in the scratch area caused by manual scratching does not influence the migration speed of the cells. Scratch introduced by two different pipette tips. **(B)** Which can be corrected by calculating the change in tissue area over time.


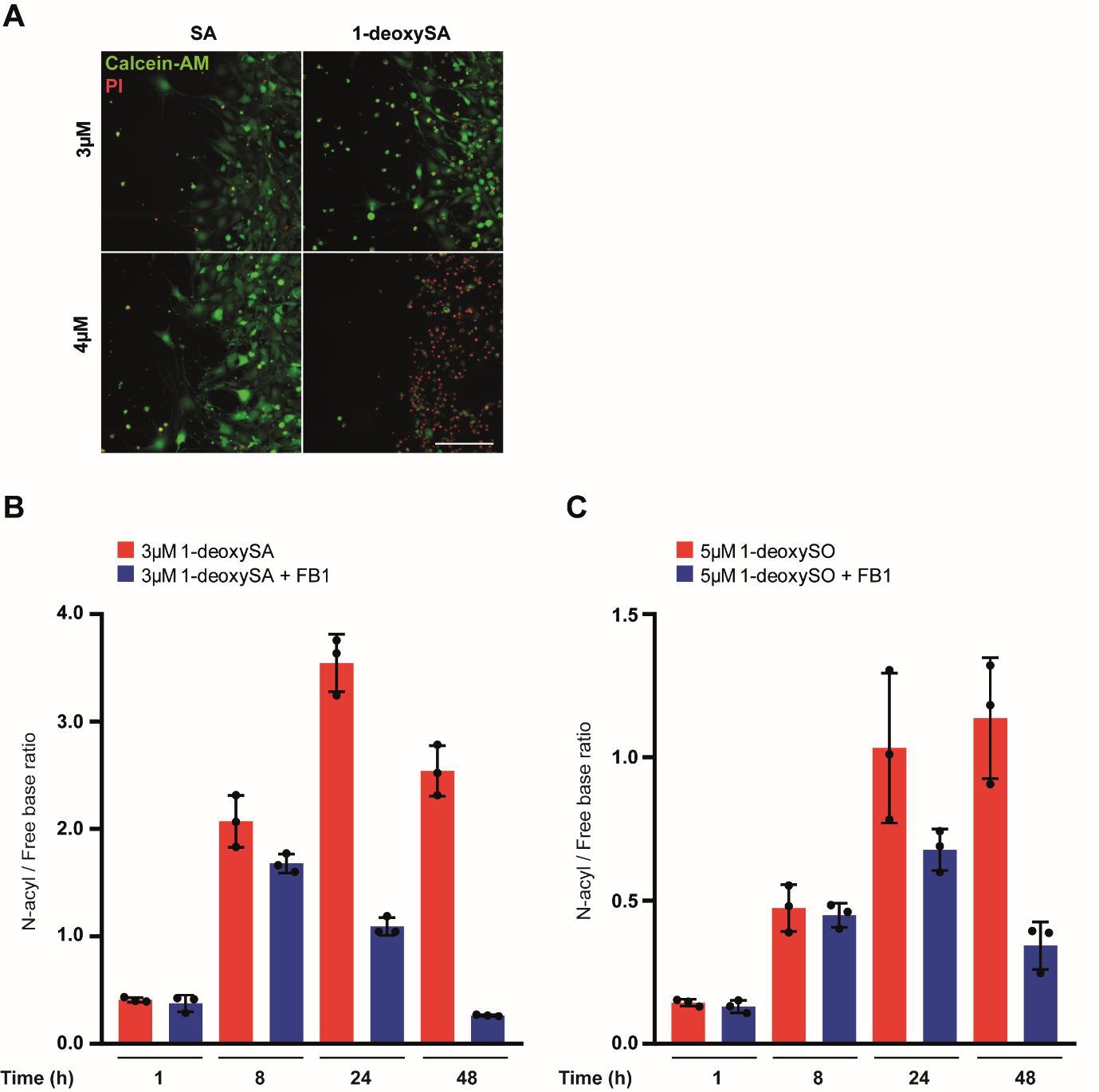


**Supplementary Figure 2. (A)** Survival of cells supplemented with SA and 1-deoxSA. Cells treated with 1-deoxySA or SA at 3µM were alive whereas 4 µM 1-deoxySA was lethal. Live cells cleave Calcein AM (Green) and are impermeable to Propidium Iodide (red). Scale bar, 100 µm. **(B-C)** LC-MS/MS analysis of NIH-3T3 fibroblast supplemented with LCBs ± FB1 (7µM) for 48 hours. **(B)** FB1 (7µM) decreased the N-acylated/free base ratio of 1-deoxySLs in 1-deoxySA treated cells after 24 hours **(C)** Similar trend was observed in cells treated with 1-deoxySO^Δ14Z^ in combination with FB1. Error bars indicate the mean ± SD. Data is representative of at least three independent experiments.

**
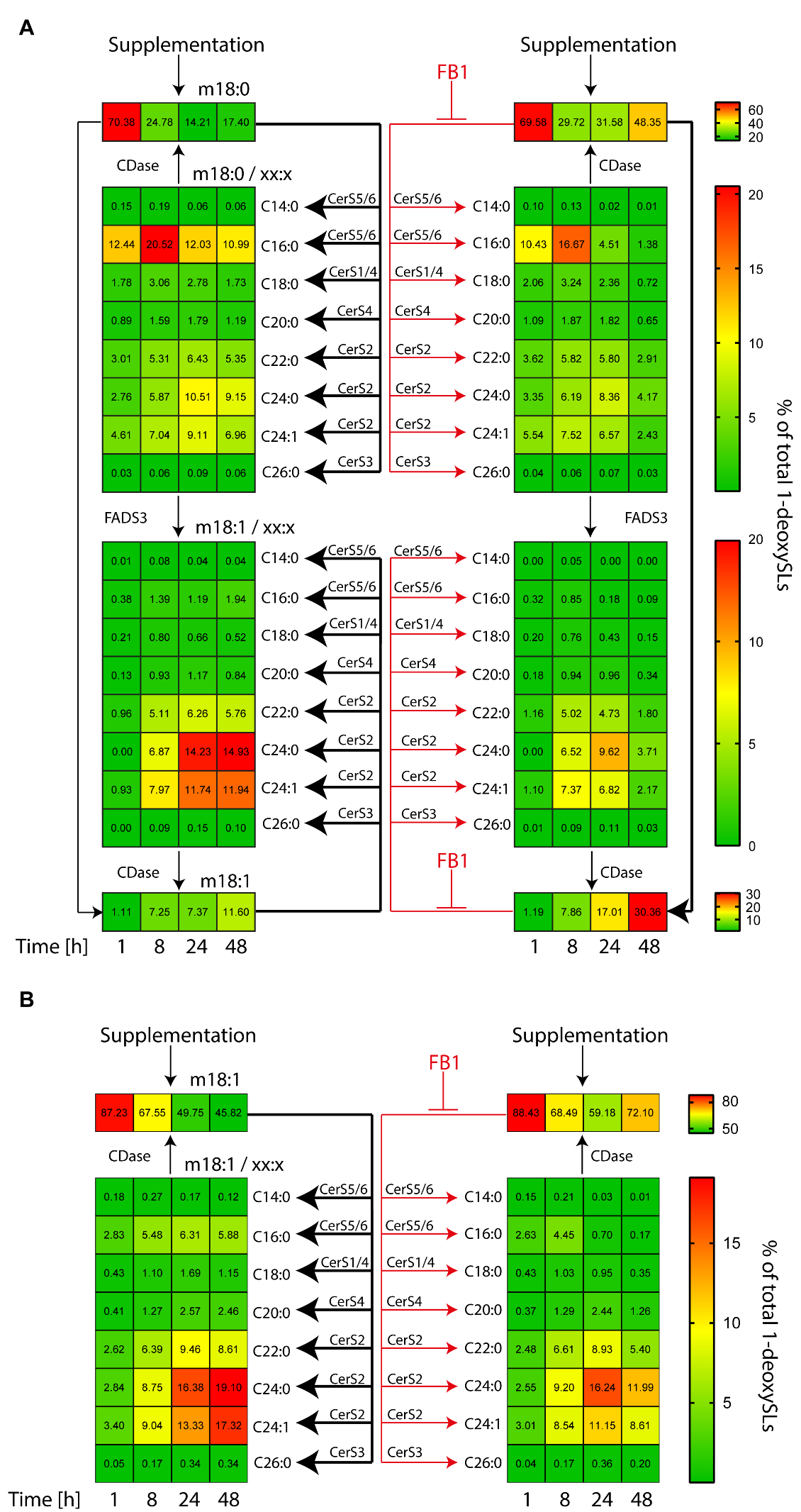
**

**Supplementary Figure 3.** Time dependent changes in the 1-deoxy(dh)Cer profile in NIH-3T3 fibroblasts treated with **(A)** (isotope labelled) 1-deoxySA (3µM) or **(B)** 1-deoxySO^Δ14Z^ either in the presence or absence of FB1 (7 µM). The data (values in the box) represent the mean of individual 1-deoxySL species, that were normalized to the sum of 1-deoxySLs, the coloring of the boxes represents the abundance of individual species within the same 1-deoxySL class (m18:0, m18:0/x:xx, m18:1/x:xx, m18:1). Data represents the average of at least three independent experiments.


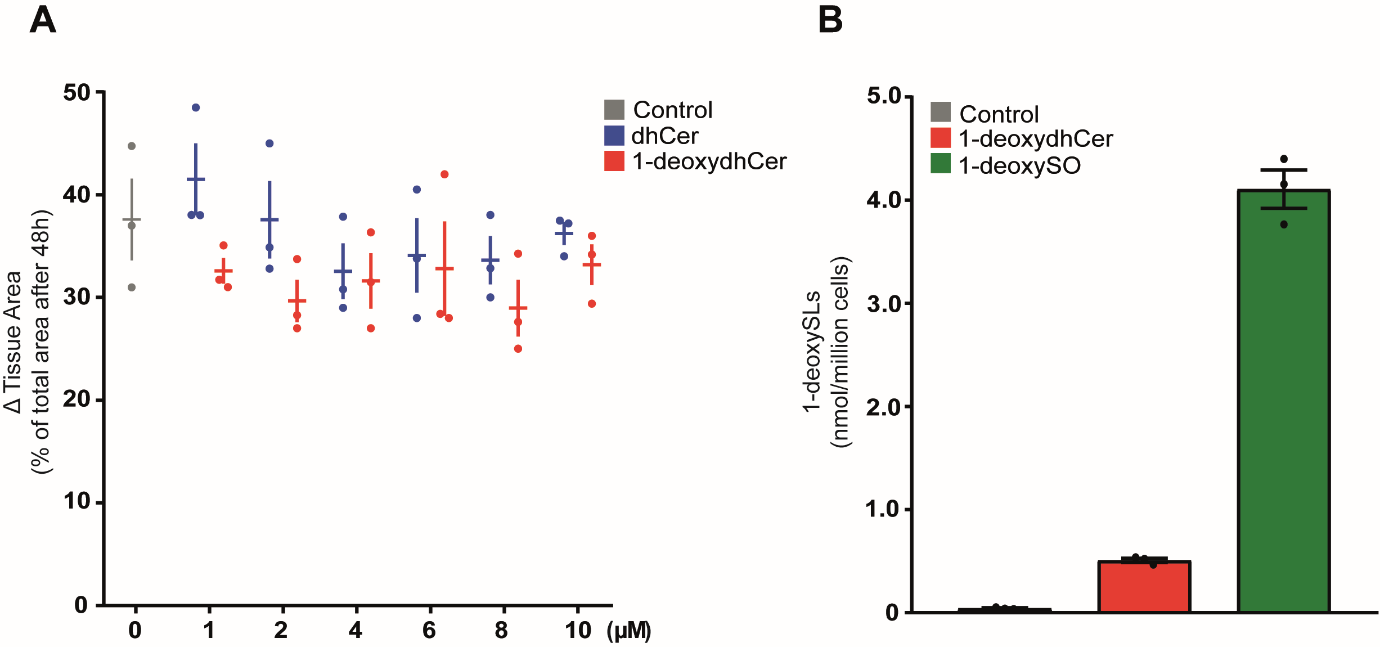


**Supplementary Figure 4.** Fibroblasts were exposed to (1-deoxy)SL treatments for 48 hours while their migration was monitored over time as described in material and methods. **(A)** Migration dose response curves of cells treated with 1-deoxydhCeramide (m18:0/24:1) or dhCeramide (d18:0/24:0) after 48 hours of incubation, no difference in the treatments compared to Control. **(B)** LC-MS/MS analysis of NIH-3T3 fibroblast treated with (1-deoxy)SLs for 48 hours. Exogenous 5 µM 1-deoxySO^Δ14Z^ (m18:1) resulted in eight fold higher 1-deoxySL levels than cells treated with the equal amount of 1-deoxydhCeramide (m18:0/24:1). Error bars indicate the mean ± SEM. Data is representative of at least six independent experiments.

**
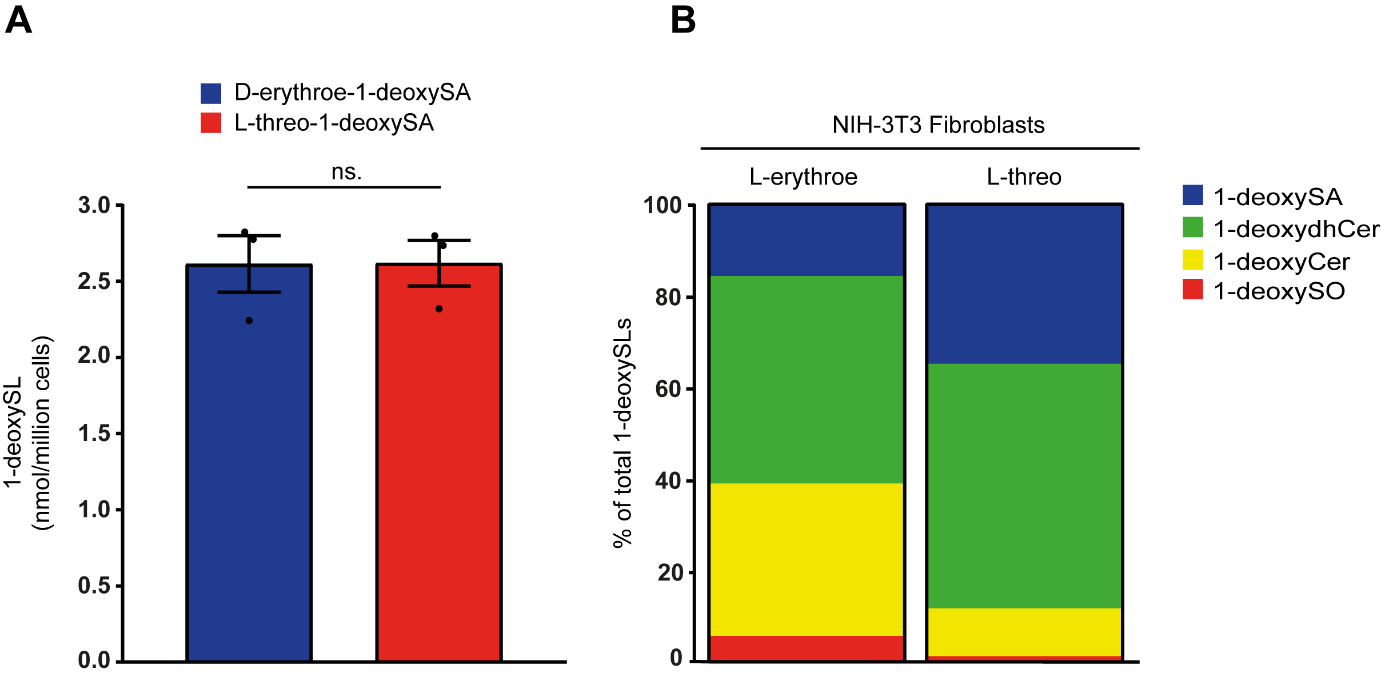
**

**Supplementary Figure 5 (A-B)** LC-MS/MS analysis of NIH-3T3 fibroblasts treated with either 3 µM D-erythro- or L-threo 1-deoxySA for 24 hours. **(A)** The total amount of 1-deoxySLs extracted from the cells is equal, **(B)** but the L-threo isoform is not desaturated equally, resulting in less 1-deoxyceramides and 1-deoxySO. Error bars indicate the mean ± SEM. Data is representative of at least three independent experiments.


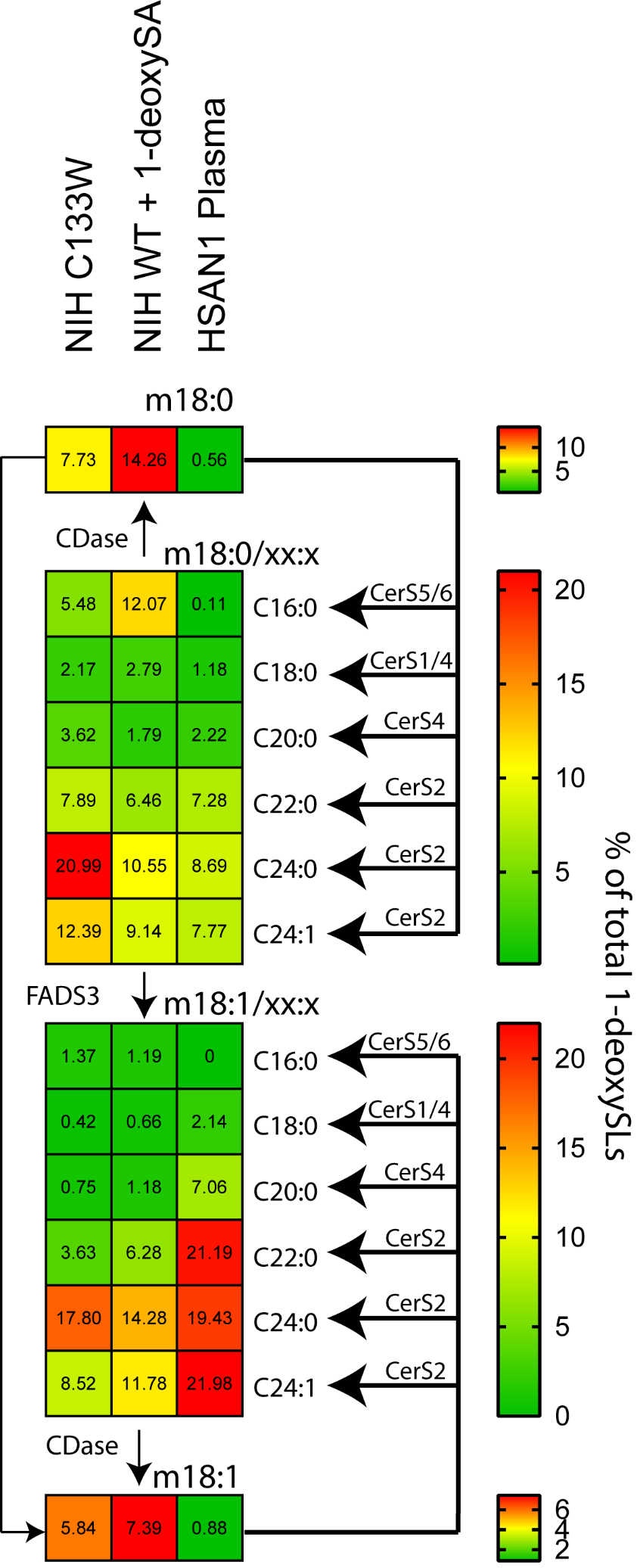


**Supplementary Figure 6** 1-deoxySL profile of mutant and WT NIH-3T3 cells, and HSAN1 plasma. Mutant cells expressing SPTLC^C133W^ were exposed Serine/Alanine challenge (see materials and methods), while WT cells were supplemented with isotope labelled 1-deoxySA for 24 hours. Comparison of labelled 1-deoxySL profiles from endogenous (originating from isotope labelled Alanine) and ectopic origin (d3-1-deoxySA supplementation) and plasma (unlabelled). In SPTLC^C133W^ cells the most abundant 1-deoxydhCer species are the very long chain species (24:0 and 24:1) while in the 1-deoxySA supplemented cells there is a marked proportion of m18:0/16:0. Distribution of 1-deoxy-ceramides is similar in both cells, where the proportion of C16:0 species is markedly reduced compared to the 1-deoxydhCer profile. Whereas in HSAN1 plasma there are almost no C16:0 species. The data (values in the box) represent the mean of individual 1-deoxySL species, that were normalized to the sum of 1-deoxySLs, the coloring of the boxes represents the abundance of individual species within the same 1-deoxySL class (m18:0, m18:0/x:xx, m18:1/x:xx, m18:1). Data represents the average of at least three independent experiments.

**Supplementary Videos**

Representative videos of cell behavior (48 hours) in the scratch assay (see materials and methods) in the presence of either sphinganine (SA), sphingosine (SO), 1-deoxysphinganine (1-deoxySA) or 1-deoxysphingosine (1-deoxySO^Δ14Z^) at concentrations indicated in the file name.

List of videos:

1. Vehicle
2. 3 µM 1-deoxySA
3. 4 µM 1-deoxySO
4. 6 µM 1-deoxySO
5. 10 µM SA
6. 10 µM SO
